# Work-related ocular events among Nigerian dental surgeons

**DOI:** 10.1186/s40557-015-0060-5

**Published:** 2015-03-20

**Authors:** Clement C Azodo, Ejike B Ezeja

**Affiliations:** Department of Periodontics, University of Benin, Benin City, Nigeria; Department of Preventive Dentistry, University of Benin Teaching Hospital, Benin City, Nigeria

**Keywords:** Ocular splashes, Foreign bodies, Work-related, Dental procedures, Dental surgeons

## Abstract

**Objective:**

Daily clinical activities in dental operatory expose dental surgeons to varied forms of ocular events. The purpose of the study was to determine the prevalence and pattern of ocular splashes and foreign bodies among dental surgeons in Nigeria.

**Methods:**

This questionnaire-based cross-sectional of dental surgeons in Southern Nigeria was conducted between September 2010 and August 2011. The information elicited were demography, experience and type of ocular event, implicated dental procedure and action taken.

**Results:**

Of the 185 studied, 148 of them responded. Of these 148 respondents, 56 (37.8%) reported foreign body, 18 (12.2%) splash, 33 (22.3%) both foreign body and splash while 41 (27.7%) reported no ocular event. It therefore means that the overall prevalence of ocular events among the respondents was 107 (72.3%). The prevalence of ocular events was significantly associated with age (p = 0.014), years of practice (p = 0.033) and safety eye goggle use (p = 0.023). The main dental procedures implicated in the ocular events among the respondents were scaling 77 (72.0%), tooth/cavity preparation 17 (15.9%), polishing 11 (10.3%) and forcep tooth extraction 10 (9.3%). The major implicated aetiological agents in the ocular events were calculus 74 (69.2%), saliva 29 (27.1%), mixed blood & saliva 19 (17.8%), tooth particles 15 (14.0%) and blood 9 (8.4%). The predominant action taken by the respondents was to rinse the eye under running water 89 (83.2%).

**Conclusion:**

Ocular splash and foreign body events are high among dental surgeons in Nigeria. Age, years of practice and safety eye goggles wear were also found to be associated with ocular events. Eye safety awareness is therefore deemed a necessity for dental surgeon in Southern Nigeria.

## Introduction

Work-related ocular injuries, arising from mechanical, chemical, microbiological, and electromagnetic insult are common and constitute a significant morbidity and disability among dentists [1-4]. Studies have shown that dentists in comparisons with other professionals in dentistry and other fields of human endeavor experience higher work-related adverse health reactions of the eyes [5,6]. These ocular injuries and conditions are relatively common among dentists in different parts of the world. About three-quarters (73.0%) of Greek Endodontists reported ocular accident experience [7]. On the average, dentists in Canada reported 1.5 mucous membrane exposures per year with the highest frequencies of blood splashes to the eyes among Oral Surgeons (1.8 per year) [8]. High prevalence of ocular injuries have been reported in dental professionals in Nigeria [9,10]. However the respondents in these Nigerian studies were recruited exclusively from a single teaching hospital with recommendations of multicenter studies.

Dental care procedures involve the use of rotary instruments and cutting and use of wires which generate foreign bodies, splatter and aerosols containing water, saliva, microorganisms, blood, tooth particles, lubricating oil, and restorative materials. These materials and fluids, play a significant role in causing injuries to the unprotected eye [11,12]. Adverse eye reactions from cold-curing acrylics and bonding materials have been reported in specialist (Orthodontic and Periodontology) dental care personnel [13,14]. Infections can be caused by splashing material, aerosols, and trauma from wires, burs, and other projectiles. Penetrating injuries from wire segments or adhesive chips during debonding and ultraviolet light of photopolymerizing units which are risk factors for cataracts, may also occur in dental practice.

Ocular injuries in dental practice may have serious and long term effect and sometimes lead to loss of vision in one or both eyes [15]. This will then impair the affected dentist future clinical practice. The effect of injury usually depends on the types of injuries which may include corneal abrasions, burns, conjunctivitis, foreign bodies and penetrating injuries. Contamination of the eye accidentally with bodily fluid (blood and saliva) carries serious potential to cause bacterial and viral infection even in the absence of eye mucosal breach or abrasion [16].

Protective eyewear during dental procedures and patient-care activities shields the eyes from splatter or debris generated but the compliance with protective eye wear among dental professionals worldwide is poor. Studies among dental surgeons have consistently shown poor adherence to eye safety practices which increases their tendencies to suffer ocular injuries [4,9]. The paucity of data on multicenter work-related ocular injuries among dental surgeons in the literature especially in developing countries, the none assessment of the association between ocular events and demographic characteristics other than gender, causes and action taken after the ocular injuries in the available unicenter studies inspired this study [8,9]. The purpose of this study was to determine the prevalence and pattern of ocular splashes and foreign bodies among dental surgeons in Southern Nigeria.

## Methods

This cross-sectional study was conducted among 185 dental surgeons of different cadres recruited from six tertiary Oral healthcare centers in Southern Nigeria between September, 2010 and August, 2011. These tertiary hospitals which are actively involved in undergraduate and postgraduate dental workforce training include University College Hospital Ibadan, University of Benin Teaching Hospital Benin City, University of Nigeria Teaching Hospital Enugu, University of Port Harcourt Teaching Hospital Port Harcourt, Lagos University Teaching Hospital Idi Araba and Obafemi Awolowo University Ile-Ife.

A pretested self-administered questionnaire designed by the authors was used for data collection.

The questionnaire was test and re-tested on ten dental surgeons working in private and secondary health facilities in Benin-City in a four week interval with reliability Cronbach's alpha of 0.85. The information elicited were demography, experience and type of ocular events in the last 12 months, procedure during which it occurred and action taken. The ocular events that involved fluid were categorized as ocular splash while those that involved solid substances were categorized as ocular foreign body.

Ethical approval was obtained from the College of Medical Sciences, University of Benin, Benin-City, Nigeria Research and Ethics Committee. Informed consent was obtained from all the research participants. Data was subjected to frequencies, percentages, cross tabulation using Statistical Package for Social Sciences (SPSS) version 17.0. The test for significance was done using Chi square statistics and P < 0.05 was considered significant.

## Results

Out of the 185 questionnaires, 148 questionnaires were returned giving an overall response rate of 80.0%. Dentists older than 30 years constituted 69 (46.6%) of the respondents. The male: female ratio of the respondents was 1.4:1.0. A total of 63 (42.6%) of the respondents reported treating more than three patients daily. Of the 148 respondents, 56 (37.8%) reported foreign body, 18 (12.2%) splash, 33 (22.3%) both foreign body and splash while 41 (27.7%) reported no ocular event. It therefore means that the overall prevalence of ocular splashes and foreign body among the respondents was 107 (72.3%). The prevalence of ocular events was significantly associated with age and years of practices. The pattern of safety eye goggle wear among the respondents were never 32 (21.6%), rarely 37 (25.0), occasionally 29 (19.6%), sometimes 39 (26.4%) and always 11 (7.4%). The prevalence of ocular events was significantly associated pattern of safety eye goggle wear (Table 1). The main dental procedures implicated in the ocular events among the respondents were scaling 77 (72.0%), tooth/cavity preparation 17 (15.9%), polishing 11 (10.3%) and forcep tooth extraction 10 (9.3%) (Table 2). The implicated aetiological agents in the ocular events were calculus 74 (69.2%), saliva 29 (27.1%), mixed blood & saliva 19 (17.8%), tooth particles 15 (14.0%), blood 9 (8.4%), amalgam particles 6 (5.6%), polishing paste 5 (4.7%), acrylic 4 (3.7%), normal saline 4 (3.7%), wire 3(2.8%), chlorhexidine 1 (0.9%), local anaesthetic solution 1 (0.9%) and aerosols 1 (0.9%) The predominant actions taken by the respondents were to rinse the eye under running water 89 (83.2%) and use of handkerchief to clean my eye 18 (16.8%). Others actions were somebody blew the eye 9 (8.4%), rubbed the eye with back of gloved hand 5 (4.8%), used eye drop to prevent infection 4 (3.7%), visited the eye clinic 6 (5.6%) and went for HIV testing 4 (3.7%) (Table 3).

**Table 1:** **The prevalence of ocular events and it’s relationship with pattern of safety eye goggle use**

**Ocular events**							
	**None**	**Foreign body**	**Splash**	**Both**	***×*** ^**2**^	**P-value**	
	**n (%)**	**n (%)**	**n (%)**	**n (%)**			
Characteristics							
Age (years)					10.62	0.014	
≤30	27 (34.2)	34 (43.0)	7 (8.9)	11 (13.9)			
>30	14 (20.3)	22 (31.9)	11 (15.9)	22 (31.9)			
Gender					4.24	0.237	
Male	21 (24.1)	31 (35.6)	14 (16.1)	21 (24.1)			
Female	20 (32.8)	25 (41.0)	4 (6.6)	12 (19.7)			
Years of practice					8.76	0.033	
≤5	33 (30.0)	45 (40.9)	14 (12.7)	18 (16.4)			
>5	8 (21.1)	11 (28.9)	4 (10.5)	15 (39.5)			
Medicated glasses					0.11	0.991	
Yes	13 (27.7)	17 (36.2)	6 (12.8)	11 (23.4)			
No	28 (27.7)	39 (38.6)	12 (11.9)	22 (21.8)			
Average number treated patients per day					0.77	0.858	
≥3	24 (28.2)	34 (40.0)	10 (11.8)	17 (20.0)			
>3	17 (27.0)	22 (34.9)	8 (12.7)	16 (25.4)			
Receipt of hepatitis-B vaccination					2.50	0.475	
Yes	30(25.9)	43 (37.1)	14 (12.1)	29 (25.0)			
No	11 (34.4)	13 (40.6)	4 (12.5)	4 (12.5)			
Total	56 (38.4)	18 (12.2)	33 (22.3)	41 (27.7)			
Safety eye goggle use							
Never	10 (17.9)	4 (22.2)	8 (24.2)	10 (24.4)	32 (21.3)	23.65	0.023
Rarely	18 (32.1)	3 (16.7)	12 (36.4)	4 (9.8)	37 (25.0)		
Occasionally	10 (17.9)	6 (33.3)	1 (3.0)	12 (29.3)	29 (19.6)		
Sometimes	16 (28.6)	2 (11.1)	11 (33.3)	10 (24.4)	39 (26.4)		
Always	2 (3.6)	3 (16.7)	1 (3.0)	5 (12.2)	11 (7.4)		
Total	56 (100.0)	18 (100.0)	33 (100.0)	41 (100.0)	148 (100.0)		

Both = Foreign body & Splash.

**Table 2:** **The procedure during which ocular event occurred among the respondents**

**Procedure**	**n (%)**
Scaling	77 (72.0)
Tooth preparation	17 (15.9)
Polishing	11 (10.3)
Forcep extraction	10 (9.3)
Surgical extraction	6 (5.6)
Amalgam removal	6 (5.6)
Trimming of denture	6 (5.6)
Cutting interdental wire	5 (4.8)
Biopsy	3 (2.8)
Oral examination	2 (1.9)
Irrigation	2 (1.9)
Cutting orthodontic wire	1 (0.9)
Suturing	1 (0.9)
Root planing/curettage	1 (0.9)
Surgical procedure	1 (0.9)

**Table 3:** **Measures taken on occurrence of the ocular events among the respondents**

**Action taken**	**n (%)**
Rinsed eyes under running water	89 (83.2)
Somebody blew the eye	9 (8.4)
Rubbed the eye with back of gloved hand	5 (4.8)
Used handkerchief or tissue to clean my eye	18 (16.8)
Used eye drop to prevent infection	4 (3.7)
Visited the eye clinic	6 (5.6)
Went for HIV testing	4 (3.7)

## Discussion

In oral health delivery services, dental surgeons are burdened by a lot of occupational issues which may be physical, biological, chemical and psychological in nature qualifying dental practice, as a dangerous and very stressful profession. It is therefore not surprising that about three-quarters (72.3%) of the respondents reported ocular event experience in this study. This was comparable to 73.0% ocular accidents reported by Zarra and Lambrianidis [7] among Greek Endodontists but higher than 60.0% reported among Jordanian dentists [15]. This high prevalence of ocular event in this study indicates that the eyes of dentists in Southern part of Nigeria are experiencing preventable harm and therefore need to be protected. The pattern of the ocular events were foreign body (37.8%), splash (12.2%), both foreign body and splash (22.3%). It is known that mucocutaneous exposures to blood and body fluid are high in dental practice with annual prevalence report of 29.0% with half of them affecting the eyes [17]. The prevalence of the ocular foreign body in this study was cumulatively higher than the reported values in Saudi Arabia (51.0%) [4], United Kingdom (48.0%) [18] and available Nigerian studies (24.4-27.0%) [8,9]. The lower use of eye protector in this study may be the explanation for the high prevalence of ocular event as a significant relationship was found between safety eye goggle use and ocular event experience. McCarthy et al. [10] also found non-use of eye protection as a risk factor for mucous membrane exposure. The ocular events reported in this study were least among respondents that reported always wearing safety eye goggles while attending to patients. This supports the need for the regular eye goggle use among dental surgeons. In this study, protective eye wear was always worn by 7.4% of the respondents and this is lower than 11.6% reported among dentists studied in 3 states in South-western areas of Nigeria [19] and 23.1% reported among dentists studied in 4 states in South-western and South southern areas of Nigeria [20]. However it is higher than 4.8% reported among dentists engaged in active clinical practices in public hospitals in Lagos, Nigeria [21]. This contrast with the findings of previous studies may be explained by the geographical difference in infection control practice which safety eye goggle wear is a component [22].

The implication of blood and body fluids [saliva (27.1%), blood (8.4%) and mixed blood & saliva (17.8%) as aetiological agents in the ocular splashes showcase the possibility of attending dental surgeons contracting infection from infected patient through the ocular mucosa. This is against the backdrop that accidentally contamination of the eye with bodily fluid (blood and saliva) in the absence of breach and abrasion carries serious potential to cause bacterial and viral infections [16]. Control and prevention can be actualized by the improvement of eye safety wear and precautionary work etiquette. Older dentists and those that have practiced for the more than the five years reported more ocular events than their counterparts. This contrasted with findings in other professions where the young and the less experienced suffered more occupational ocular injuries [23,24]. This higher prevalence among the older and more experienced dentists in this study probably reflects either their less occupational safety consciousness with time due to their queries about the evidence base of these occupational safety practices or their performance of more complex procedures thereby increasing susceptibility. Al-Dharrab and Al-Samadani [25] reported that younger dentists follow safer practice and are more careful in using protective wear than older ones and Tada et al. [26] reported poorer compliance with infection control practices in older than younger dentists.

The most common dental conditions in Nigeria are periodontal disease and dental caries. They are managed by scaling and polishing, cavity preparation for restoration and extraction for the non-restorable and complicated carious cases. This is the obvious explanation of why the main dental procedures implicated in the ocular events were mainly scaling, tooth/cavity preparation, polishing and forcep extraction. However, the majority of actions taken among the respondents with ocular events in this study were restricted to the dental clinic. The predominant action taken was to rinse the eye with water which may have helped to give relief to the affected dental surgeon because calculus and saliva were main aetiological ocular foreign body and splash respectively. Anecdoctally, some of the behaviours were not different from common behaviours of non-professional with foreign body in their eye. Self-medication with eye drop to prevent infection was also reported 3.7% of the respondents. However some actions that took place outside the dental clinic includes eye clinic visit (5.6%) and Human Immunodeficiency virus (HIV) testing (3.7%). This highlighted either a heightened health and ocular concern or the possibility of severe injury that trigger proper ophthalmological care seeking and fear of mucosal HIV transmission. Although, this study finding may be limited by recall bias, the comprehensiveness of the ocular event in relation to foreign body and splash in this study will be an obvious justification for further onsite study.

## Conclusion

Data from this study revealed a high prevalence of ocular events among dental surgeons in Southern Nigeria. Age, years of practice and safety eye goggles wear were also found to be associated with ocular events. Eye safety awareness is therefore deemed a necessity for dental surgeon in Southern Nigeria.
